# Fine tuning and optimization of magnetic hyperthermia treatments using versatile trapezoidal driving-field waveforms

**DOI:** 10.1039/d0na00358a

**Published:** 2020-09-01

**Authors:** Gabriele Barrera, Paolo Allia, Paola Tiberto

**Affiliations:** INRIM, Advanced Materials Metrology and Life Sciences Strada delle Cacce 91 I-10135 Torino Italy g.barrera@inrim.it +39 011 3919858

## Abstract

Applying trapezoidal driving-field waveforms to activate magnetic nanoparticles optimizes their performance as heat generators in magnetic hyperthermia, with notable advantages with respect to the effects of harmonic magnetic fields of the same frequency and amplitude. A rate equation approach is used to determine the hysteretic properties and the power released by monodisperse and polydisperse magnetite nanoparticles with randomly oriented easy axes subjected to a radio-frequency trapezoidal driving field. The heating ability of the activated nanoparticles is investigated by means of a simple model in which the heat equation is solved in radial geometry with boundary conditions simulating *in vivo* applications. Changes of the inclination of the trapezoidal waveform's lateral sides are shown to induce controlled changes in the specific loss power generated by the activated nanoparticles. Specific issues typical of the therapeutic practice of hyperthermia, such as the need for fine tuning of the optimal treatment temperature in real time, the possibility of combining sequential treatments at different temperatures, and the ability to substantially reduce the heating transient in a hyperthermia treatment are suitably addressed and overcome by making use of versatile driving fields of a trapezoidal shape.

## Abstract 2

Magnetic hyperthermia healing treatments are optimized and tuned by exploiting trapezoidal driving fields.

## Graphical abstract

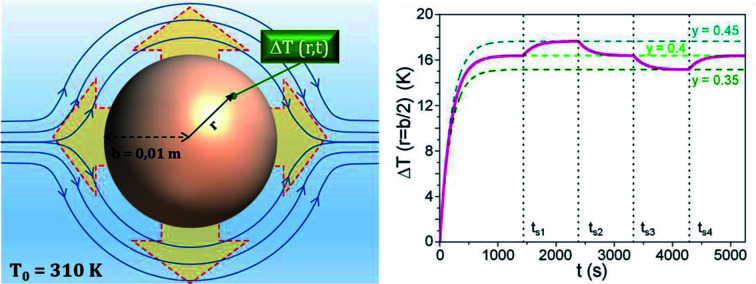

## 1 Introduction

Magnetic hyperthermia and related heat-assisted healing treatments are nowadays one of the most widely studied areas of application of magnetic nanoparticles (NPs).^1–7^ Precision nanomedicine is a modern therapeutic practice, mainly developed for the treatment of cancer, and it exploits nanotechnology to support and favour treatments aimed at healing patients in a non-invasive manner.^8–10^ The important role played by magnetic NPs in precision nanomedicine is widely recognized: particles can act as point-like heating agents, can be targeted towards the malignant tissue and can diffuse around or within the small region subjected to treatment.^11–13^

Magnetic hyperthermia is sometimes exploited as a standalone cure for cancer^5,11,13–15^ aimed to selective killing of malignant cells (tumor apoptosis)^5,13^ or complete tissue necrosis by the ablation process.^16,17^ In recent years, magnetic hyperthermia has been increasingly combined with other anti-tumor therapies, such as chemotherapy and radiotherapy, resulting in an enhancement of both therapeutic efficacy^11,13,18–24^ and tumor penetration.^25^

In spite of the vast literature on this subject area, magnetic hyperthermia is still not completely understood nor completely optimized. In fact, application of the technique to therapeutic practice poses a great number of intertwined problems pertaining to different fields such as physics, chemistry, engineering and medicine.^26^

Optimization of hyperthermia treatments clearly requires, as a necessary condition, optimization of the mechanism of heat release from magnetic NPs, in order to maximize their specific loss power (SLP),^6,27–29^ defined as the total power released by the magnetic nanoparticles divided by their total mass. This can be done by either looking for higher-performance magnetic nanomaterials^30,31^ and better particle sizes and shapes,^32,33^ or trying to devise methods to more efficiently extract the heating power from a given system of nanoparticles.^28^

In most *in vitro* and *in vivo* applications, the particles are activated by using a radio-frequency (RF) harmonic magnetic field.^14,34^ However, it has been recently shown that the sinusoidal waveform is not always the best choice, and that controlling the shape of the driving-field waveform may result in a significant enhancement of the SLP of magnetic NPs evenly distributed in a host medium.^28^ In fact, choosing a suitable magnetizing waveform not only has an effect on the SLP, but can also solve a number of practical problems arising in the therapeutic practice.

For instance, in hyperthermia treatments, the therapeutically effective temperature interval has to be reached with precision, which is a not easy task in the *in vivo* practice where a number of ill-controlled parameters, many of which are related to the natural but unpredictable differences existing from body to body, act to jeopardize the achievement of an optimal therapeutic efficacy.^9^ Having the possibility of adjusting the steady-state temperature of the treated region in real time (*i.e.*, without interrupting the treatment to recalibrate the volume fraction of inoculated particles) would represent a big step forward.

When magnetic hyperthermia is used for tumor apoptosis, the necessity of avoiding damage of healthy tissues poses a strict upper limit (typically, 41–43 °C (ref. 36 and 37)) to the steady-state temperature which has to be maintained in the target region for a rather long time (tens of minutes, up to about one hour^38^). On the other hand, in the case of heat-assisted tumor ablation resulting in tissue necrosis, much higher temperatures (usually more than 60 °C and up to 70–80 °C) need to be reached in the target region for a limited time.^16,39^ In the current therapeutic practice, magnetically operated low-temperature hyperthermia and high-temperature ablation are distinct treatments which are typically performed using different magnetic nanomaterials and different particle concentrations. A possible therapeutic opportunity could be to perform a combined ablation-hyperthermia cycle, where a shorter, high-temperature treatment is followed by prolonged heating at a lower temperature, by making use of the same clinical setup and the same volume fraction of inoculated magnetic nanoparticles.

Another important aspect which requires optimization is the existence of long thermal transients between the start of a treatment (*i.e.*, when the RF magnetic field is switched on) and the time at which the working temperature is actually reached.^40^ The therapeutic practice would greatly benefit from a reduction of the initial transients.

In this paper, we show that all the aforementioned issues can be solved by activating magnetic NPs by means of a trapezoidal instead of a harmonic driving-field waveform. Trapezoidal waveforms turn out to be easy to produce and control, and sufficiently versatile to successfully address the outlined problems. Our model explicitly refers to particles of magnetite (Fe_3_O_4_) having diameters in the 10–16 nm range, because nanometer-sized magnetite has taken a prominent role in biomedical applications.^4,15^

The effect of using a trapezoidal waveform on the SLP of magnetite nanoparticles is investigated by determining the area of the hysteresis loops which appear at the magnetizing frequency. The loops are obtained using a rate-equation approach, which is able to suitably describe the dynamics of magnetization in NPs subjected to an alternating field of high frequency while maintaining intrinsic simplicity and effectiveness.^41^

The heating efficacy of a set of magnetite nanoparticles subjected to a trapezoidal driving field waveform is finally evaluated by making use of a simple heating model to picture a small portion of living tissue. The proposed technique of nanoparticle activation by using trapezoidal waveforms is shown to give a substantial contribution to the optimization of magnetic hyperthermia.

## 2 Magnetite nanoparticle activation using trapezoidal magnetic-field waveforms

### 2.1 Magnetic nanoparticles as double well systems (DWSs)

The properties of magnetic nanoparticles are well described by a simple model where the key role is played by the energy barrier separating two local energy minima. Therefore, magnetic nanoparticles can be assimilated to classical double-well systems (DWSs)^42,43^ with a barrier originating from magnetic anisotropy. Describing nanoparticles as DWSs involves a number of implicit assumptions, in particular that each nanoparticle behaves like a macrospin^44^ and that the effective magnetic anisotropy energy has uniaxial symmetry.^45^ In spite of all approximations,^46^ the model has proven to account for most experimental observations, and to provide an adequate picture of the main features of magnetic nanoparticles, particularly the ones of higher interest in applications.^45^

Magnetite nanoparticles are treated as non-interacting DWSs described by three parameters: magnetization *M*_s_, magnetic anisotropy constant *K*_eff_, and size *D*. The magnetic moment of each particle is *μ* = *M*_s_*V* where 
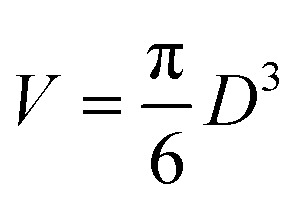
 is the particle volume; the energy barrier separating the two energy wells is *E*_B_ = *K*_eff_*V*.

Considering nanoparticles as non-interacting is of course an approximation, and in some cases it may be an oversimplification.^47–52^ The assumption is however reasonable in the light of the ultimate aim of the paper, which is to highlight the advantages of applying a non-conventional driving field waveform on the SLP of a set of dispersed nanoparticles, independent of their degree of interaction. It should be noted that in the current therapeutic practice there is a tendency towards the reduction of the concentration of inoculated magnetic particles (associated with optimization of their SLP),^11^ in order to minimize potentially negative effects on the patient's body.^4^ Let us finally stress that the effective anisotropy constant *K*_eff_ can incorporate, although in an approximate manner, weak interparticle interactions,^43,53^ thereby allowing a collective effect to be reduced to a single-particle picture.

The room-temperature values of the magnetic parameters used in this work are *M*^RT^_s_ = 350 emu cm^−3^ and *K*^RT^_eff_ = 4 × 10^5^ erg cm^−3^, in line with those in the literature.^54–56^ However, in the application of magnetite nanoparticles to magnetic hyperthermia, the temperature dependence of both magnetization and magnetic anisotropy cannot be neglected.^35^ In this paper, the temperature behaviour of *M*_s_ and the Curie temperature (*T*_C_ = 856 K) are taken from published data.^35,57^ Uniaxial anisotropy is assumed to vary according to the third power of magnetization:^35,58^1
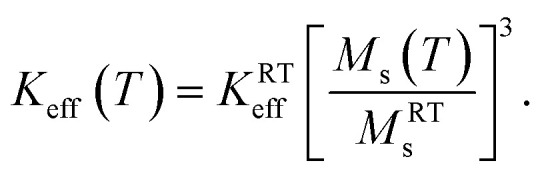


The particle diameters considered in this study are in the range 10–16 nm, corresponding to the most frequent interval of values in actual *in vitro* measurements and *in vivo* application of magnetite nanoparticles.^14,15^

### 2.2 Rate equations: advantages and limits of applications

Rate equations are being applied since about one decade to study the response of uniaxial magnetic NPs described as double-well systems (DWSs).^41,59^ The method has been successfully applied to systems of non-interacting particles, under both equilibrium^43,59^ and off-equilibrium conditions. In particular, rate equations are used to describe important off-equilibrium properties, such as the behaviour of the low-field magnetization below the blocking temperature in field cooling/zero field cooling experiments,^43^ and isothermal magnetic hysteresis loops.^28,41^

The method provides an accurate picture of the process of magnetization of an assembly of DWSs, under both linear and non-linear, static and dynamic conditions. Rate equations work well over an extended range of driving-field amplitudes and frequencies;^41^ they describe with precision the transition between superparamagnetic and blocked regimes in a nanoparticle,^43^ and are appropriate for treating the off-equilibrium response of nanoparticles subjected to a cyclic field of frequency up to hundreds of kHz (ref. 41) and of an arbitrary waveform,^28^ independent of the state of blocking of the particle.

In this respect, it should be noted that at sufficiently high driving-field frequencies a typical off-equilibrium behaviour emerges even in particles which under quasi-static conditions are well inside the reversible region (the blocking temperature of a magnetic NP is a frequency-dependent quantity,^41^ so at high magnetizing frequencies it becomes much higher that the value is appropriate to the quasi-static case); as a consequence, a hysteresis loop sustained by the operating frequency opens even in particles which are in thermal equilibrium at zero frequency. This effect is well described by the rate equations.

Finally, the rate-equation method is appropriate for studying both monodisperse and polydisperse systems of nanoparticles with easy axes randomly pointing in all directions.^41^

Of course, more exact approaches to the dynamics of magnetization in nanostructures exist. They involve solving either the Landau–Lifshitz^60^/Landau–Lifshitz–Gilbert^61^ equations, or the Fokker–Planck–Brown equation for the magnetization dynamics.^62,63^ The difficulties arising when either of these methods is adopted are briefly discussed elsewhere.^28^ As an approximation to a more complex problem, the rate-equation approach necessarily exhibits some drawbacks and has some limits of application, as discussed elsewhere.^28^ The predictions of the rate-equation method applied to magnetite nanoparticles were shown to gradually lose validity with the decreasing particle size; with the values of the magnetic parameters used in the present paper, magnetite nanoparticles should have a diameter *D* > 11 nm in order to be correctly described by the rate equations. The range of *D* values examined in this paper complies with such a requirement.

The procedure adopted in this paper involves the following steps:

- the rate equations are first numerically solved for an assembly of randomly oriented nanoparticles of the same size subjected to a dynamic magnetic field, and the instantaneous populations of the two wells are calculated for all DWSs;

- the magnetization along the field direction is calculated as a function of time from the instantaneous populations in the wells,^43^ and the hysteresis loop is generated;

- the area of the loop is calculated, and the SLP is obtained;

- in polydisperse systems described by a distribution of particle diameters, the procedure is repeated for all nanoparticle sizes and a weighted sum is generated.

An abridged description of the rate-equation model is given in the following lines. Let us consider first the subset of DWSs whose easy axes make an angle *ϕ* with the direction of the applied magnetic field. The magnetic moments continuously redistribute between the two wells according to the following rate equations:2
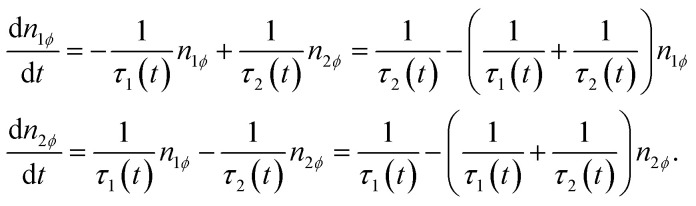


The occupancy numbers in the two wells are *N*_1*ϕ*_ and *N*_2*ϕ*_ (their sum gives the total number of DWSs of the considered subset, *N*_*ϕ*_). In eqn (2), the quantities *n*_1*ϕ*_ = *N*_1*ϕ*_/*N*_*ϕ*_ and *n*_2*ϕ*_ = *N*_2*ϕ*_/*N*_*ϕ*_ are used (*n*_1*ϕ*_ + *n*_2*ϕ*_ = 1).

The escape frequencies in eqn (2) are defined as *τ*_*i*_^−1^ = *τ*_0_^−1^ exp[−(*E*_M_ − *E*_*i*_)/*k*_B_*T*] (*i* = 1,2) where *E*_*i*_(*t*) are the energies of the two energy minima, and *E*_M_ is the energy at the top of the barrier.

The energy *E* of a single DWS of volume *V* is given by:*E* = *K*_eff_*V* sin^2^(*θ*) − *H*(*t*)*M*_s_ cos(*θ* − *ϕ*)where *θ* is the angle between the magnetic moment direction and the easy axis. The values *E*_M_ and *E*_*i*_ are found by making the derivative of *E* with respect to *θ* equal to zero and by checking the sign of the second derivative. These energy values are functions of time because in the studied case the applied field oscillates between +*H*_V_ and −*H*_V_ and *vice versa*. Note that the relation *τ*_1_(−*H*) = *τ*_2_(*H*), valid at all angles *ϕ*, is derived from symmetry considerations.

When the system is subjected to a harmonic magnetic field *H*(*t*) = *H*_V_ cos(2π*ft*), the sweep rate of the magnetic field is not a constant; therefore, a rms value is typically used in the calculations. By introducing the dimensionless field *h* = HM_s_/2*K*_eff_, the rms sweep rate is simply defined as 
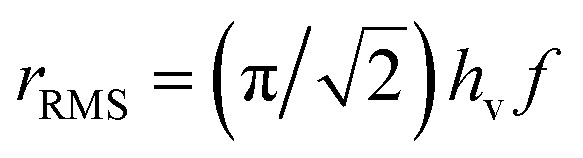
 where *h*_v_ is the dimensionless vertex field: the quantity *h* is observed to evolve from the upper (*h*_v_) to the lower vertex (−*h*_v_) and *vice versa* according to the linear law *h*(*t*) = ∓*h*_v_ ± *r*_RMS_*t*. As a consequence, the rate equations can be transformed taking *h* as the independent variable:3
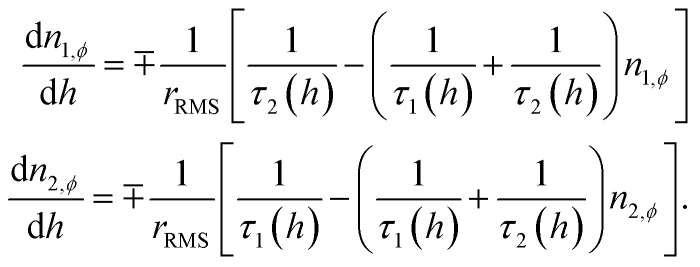
where the ∓ sign refers to the upper/lower loop branch. It should be explicitly remarked that using a rms sweep rate for a harmonic driving field waveform is perfectly equivalent to applying a triangular symmetric waveform.

Once *n*_1,*ϕ*_ and *n*_2,*ϕ*_ are obtained, the magnetization is easily found;^43^ the results for each *ϕ* angle are then easily summed up assuming a uniform distribution of easy axes.

### 2.3 Nanoparticle activation by using trapezoidal magnetic field waveforms

In theoretical and experimental studies of magnetic hyperthermia, nanoparticles are usually subjected to sinusoidal driving fields (although in fact, most of the expressions given in the literature and attributed to the sinusoidal waveform are more appropriate to the case of the triangular symmetric waveform, as explained elsewhere^28^ and recalled in the previous subsection). However, changing the waveform's type has important consequences on the thermal power generated by a system of magnetic NPs with random easy axes.^28^ In particular, the best results in terms of specific loss power (SLP)^27^ may be obtained by applying the square driving-field waveform.

**Fig. 1:**
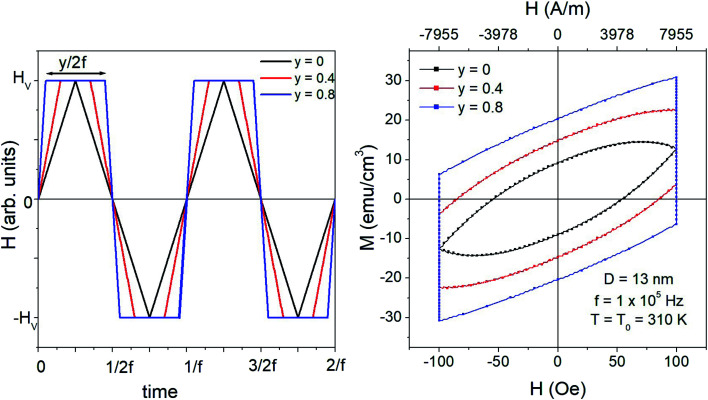
Left panel: parameters defining the trapezoidal driving-field waveform. Right panel: hysteresis loops of monodisperse magnetite nanoparticles (*D* = 13 nm) with random easy axis directions for the same values of the parameter *y* as in the left panel.

Both triangular symmetric and square waveforms are limiting cases of the general trapezoidal waveform, sketched in the left panel of Fig. 1. Trapezoidal waveforms are characterized by three parameters: amplitude (or *vertex field*) *H*_V_, frequency *f*, and taper parameter *y*. The latter quantity is a measure of the inclination of the two lateral sides of the trapezoid and is univocally related to the duration of the time elapsed at constant applied field (±*H*_V_); it is easy to show that for a wave of frequency *f*, such a duration is equal to *y*/2*f* (see Fig. 1, where the waveforms corresponding to three values of *y* are reported). Triangular and square waveforms correspond to the limiting cases *y* = 0 and *y* = 1, respectively.

**Fig. 2:**
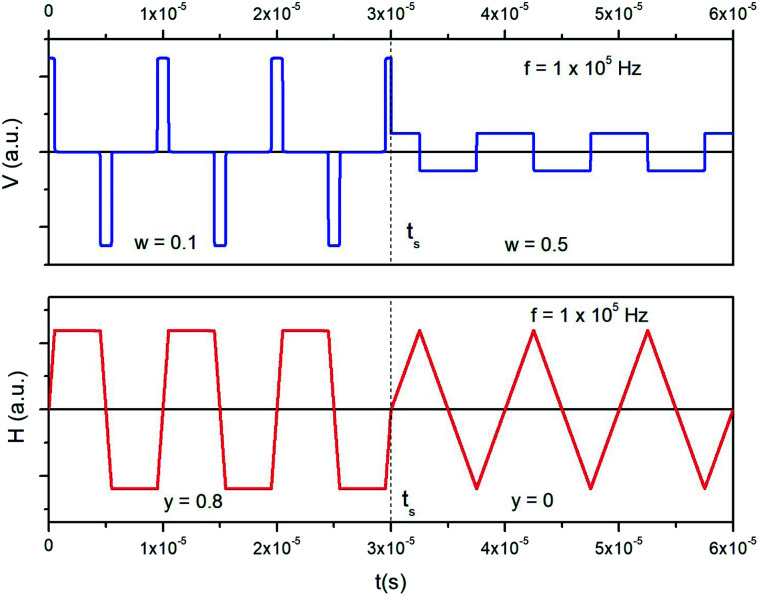
Top panel: pulsed voltage waveform at a frequency of 1 × 10^5^ Hz. Pulse duration and amplitude are changed at *t* = *t*_s_. Bottom panel: resulting electrical current waveform flowing in an inductive circuit.

A trapezoidal waveform of magnetizing electrical current can be produced by sending a rectangular input voltage waveform of a tunable pulse width and amplitude in an inductive circuit (coil). The voltage pulse width is univocally related to the shape of the taper parameter *y* of the trapezoidal wave of current. By defining the dimensionless pulse width as *w* = *D*_P_*f* where *D*_P_ is the pulse duration, one gets: *y* = −2*w* + 1; the taper parameter *y* takes values between 1 and 0 for *w* taking values between 0 and 0.5. As an example, a train of alternating voltage pulses generated in the feed circuit at the frequency *f* = 1 × 10^5^ Hz is shown in the upper panel of Fig. 2. At a given time (*t*_s_) both pulse width and pulse amplitude are suddenly modified. In this way, the waveform of the output current flowing in the coil, obtained by Fourier series analysis (lower panel) changes at *t*_s_ from trapezoidal with *y* = 0.8 to triangular (*y* = 0), maintaining the same amplitude. Therefore, a quick change of the taper parameter *y* is easily achieved when needed.

In a trapezoidal waveform, the applied field takes a constant value twice per cycle, and is quickly reversed at a constant rate twice per cycle. The time taken by each field reversal is 
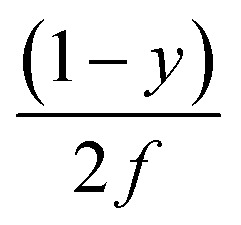
; as a consequence the absolute value of the time derivative of the magnetic field (during reversal) is 
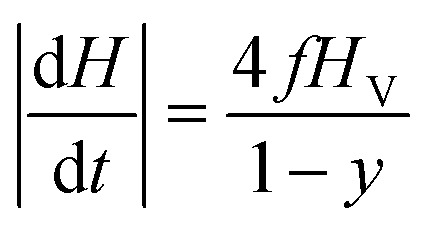
; note that for a nearly square wave (*y* → 1) the value of this derivative becomes very large, so particular precautions need to be taken in order to avoid potential damage to living tissues by the effect of the eddy currents generated by the strong magnetic flux variation.^28^ The hysteresis loops obtained at *T* = *T*_0_ = 310 K by solving the rate equations for a monodisperse assembly of non-interacting, randomly oriented magnetic nanoparticles with *D* = 13 nm are shown in the right panel of Fig. 1 for the same *y* values as in the left panel; the frequency is *f* = 1 × 10^5^ Hz and the field amplitude is *H*_V_ = 100 Oe ≃ 8 × 10^3^ A m^−1^. The product (*H*_V_*f*) is therefore well below the upper limit for biological safety proposed by Dutz and Hergt.^27^

For a triangular waveform (*y* = 0) the usual almond-like shape of a minor hysteresis loop is obtained (magnetic saturation is achieved in this case at much higher vertex fields, *H*_V_ > 1 × 10^3^ Oe). The loop's shape changes when a trapezoidal waveform is applied: in this case, the loop is characterized by two vertical segments where the magnetization, initially out of equilibrium, relaxes toward the equilibrium conditions at constant field.^28^ The other two branches of the loop correspond to quick reversal of the magnetic field. When *y* → 1, these two branches become increasingly similar to straight lines (and become nearly adiabatic^28^). The loop's area and consequently the power released by the nanoparticles at the frequency *f* monotonically increase with *y*. Similar results can be obtained for all the values of the nanoparticle diameter *D*.

More details about the effect of the taper parameter *y* on the loop's shape and on the temperature behaviour of the power *P*_in_ released by the magnetic nanoparticles are given in the following subsection.

### 2.4 Hysteresis loops and power released by the nanoparticles

**Fig. 3:**
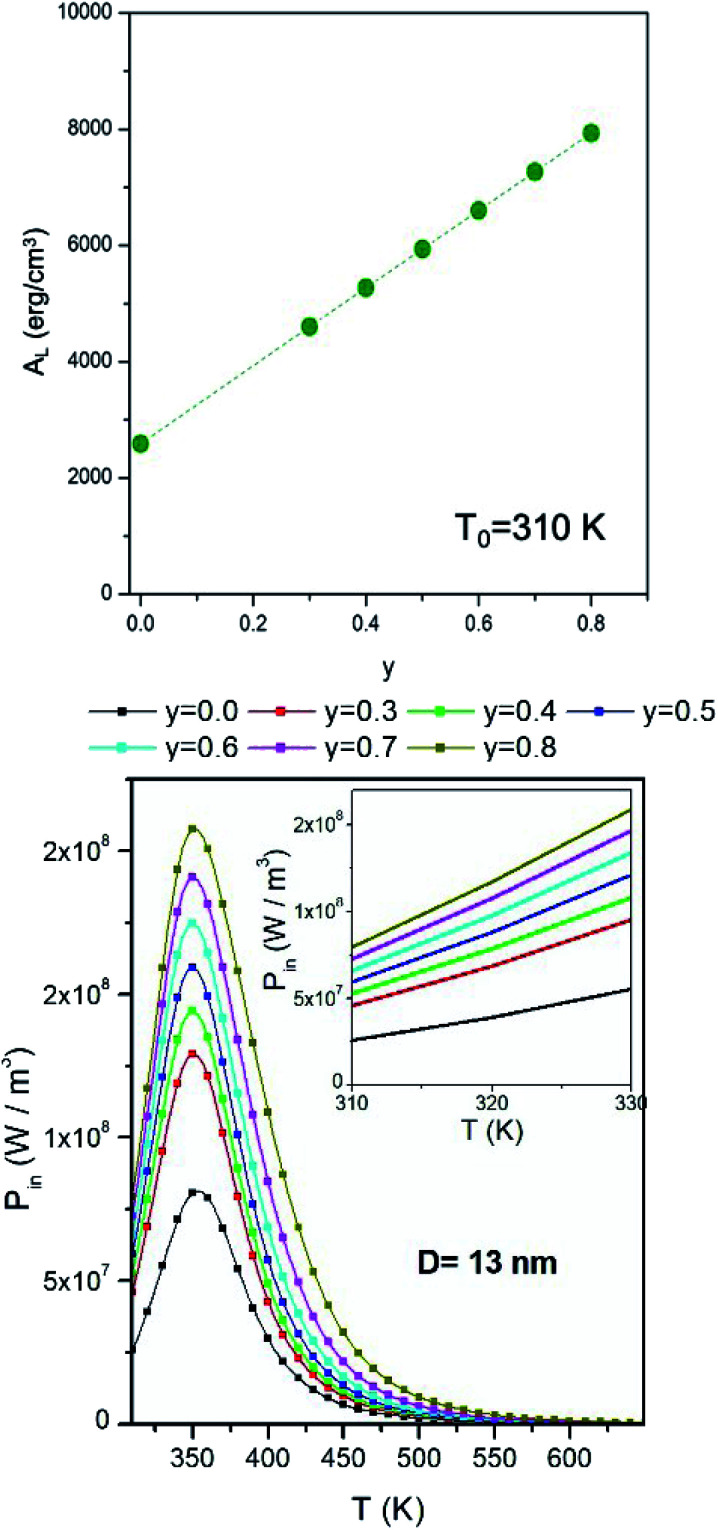
Upper panel: effect of the taper parameter on the area of the hysteresis loops of monodisperse magnetite nanoparticles (*D* = 13 nm). Lower panel: temperature behaviour of the heating power generated by the nanoparticles; the curves are reported for temperatures higher than *T*_0_ = 310 K. The inset shows the detail of the region immediately above *T*_0_.

The effect of taper parameter *y* on the hysteresis loop's area *A*_L_ at fixed temperature (*T*_0_ = 310 K) is shown in the upper panel of Fig. 3 for a monodisperse system of randomly oriented magnetite nanoparticles (*D* = 13 nm) subjected to a driving field of frequency *f* = 1 × 10^5^ Hz and amplitude *H*_V_ = 100 Oe. An almost perfectly linear behaviour of *A*_L_ with *y* is observed. As a consequence, the released power *P*_in_ = *A*_L_*f* increases in the same way when *y* is increased. When the temperature of the system of nanoparticles is raised above the starting temperature *T*_0_, the released power changes, as shown in the right panel of the same figure (in SI units). The non-monotonic behaviour of the *P*_in_(*T*) curves is related to the variation of the magnetic parameters *M*_s_ and *K*_eff_ with temperature^35^ (eqn (1)).

The maximum of the *P*_in_ curves occurs where the loop area is the largest,^35^ this condition roughly corresponding to the temperature where the typical time of jump across the DWS barrier *τ*(*T*) becomes equal to half a period of the driving field. On the other hand, the effect of increasing the taper parameter *y* is limited to an almost rigid increase of the whole *P*_in_(*T*) curve. The inset in the lower panel of Fig. 3 shows the details of the power curves in the region of temperatures close to *T*_0_, which is the most interesting one for the magnetic hyperthermia treatments considered in this paper.

### 2.5 Heating model

In magnetic hyperthermia, the SLP of a system of magnetic NPs is exploited to raise the temperature in a bounded region filled with a medium where the particles have been dispersed. Both *in vitro* and *in vivo* applications require an accurate prediction of the temperature behaviour in such a region.^15,38^ In magnetic hyperthermia, the problem of correctly predicting the steady-state temperature is complicated by the fact that the heating power *P*_in_ is itself temperature-dependent.^35^

Power dissipation by a system of non-interacting nanoparticles is often described in the linear response regime;^64^ however, for the driving field values most commonly used in practical applications^38^ (100–250 Oe, *i.e.* 9–20 kA m^−1^) the limits of validity of the linear theory are overcome, and the magnetic response of the system is no longer linear, as clearly shown by the rate equation approach.^28,41^ The present heating model takes in due account the true magnetic losses of typical nanoparticles under standard operating conditions. In particular, the rate equations governing the evolution of the populations in each potential-energy well of the DWS are solved for both monodisperse and polydisperse NP systems, so that the hysteresis loops and the heating power *P*_in_ are obtained as functions of temperature *T*. When a trapezoidal waveform is applied, the heating power is a function of the taper parameter too, as shown in the right panel of Fig. 1 (further details of the solutions for a monodisperse system are discussed in the previous subsection).

The subsequent step is to insert the heating power of magnetic origin in a suitable heat equation with the appropriate boundary conditions. Accurately modelling heat transport in living bodies is a very difficult task. Many variants of the bioheat transfer equation with internal power generation have been proposed;^65^ they are all aimed to describe with various approximations how heat can be generated, transported and dissipated in an extremely complex system such as a living body.

The problem is made very challenging by the variety and variability of parameters influencing magnetic hyperthermia in real living bodies, such as, variations in the blood composition and density, non-uniform blood flow, thermal interactions between blood vessels and tissues, and types of blood vessels significant for heat transfer in tissues. A number of bioheat equations specifically aimed to take into account most of these effects have been proposed, starting from Pennes' transfer equation^66^ and including the Chen–Holmes approach^67^ and the Weinbaum–Jiji–Lemons model^68^ and related modifications.^69^ Another important factor is the difference between healthy and malignant tissues when physiological properties important for heat dissipation, such as the tissue-blood perfusion rate, are considered; possible thermal consequences and related dangers have been discussed elsewhere from a physicist's viewpoint.^35^

As a matter of fact, all the mentioned approaches take origin from the classical Fourier equation, with suitable adaptations introduced to account for specific heat sources, heat transfer mechanisms and heat sinks typical of living tissues.^65^ It should be stressed that the emphasis of all the bioheat equations is more on the processes governing the way the heat is transported and dissipated in a living body rather than on the physical properties of the internal heat source, *i.e.*, the processes determining the energy deposition rate.

On the contrary, the present paper is specially aimed to provide a proof of principle of the tapered waveform technique, describing how and how much can the power released by magnetic nanoparticles be enhanced and controlled. As a consequence, we have taken the simplest possible thermal model, *i.e.*, the standard Fourier equation in radial symmetry^35,70,71^ with a distributed heat source and with boundary conditions simulating heat loss dominated by forced convection, a common situation in living bodies where the excess heat generated in a small region is basically taken away by blood flow.^65^ Therefore, our results can be of interest not only for *in vitro* experiments, but also for *in vivo* healing treatments.

Focussing on a particularly simple heat equation and simplified boundary conditions is fundamental to easily grasp features and advantages of a technique aimed at enhancing the magnetic response of nanoparticles and their thermal performance, as well as at providing the final user with a better control of the heating process.

Of course, the predictions of the present proof of principle may become more accurate by inserting the power released by nanoparticles in a bioheat equation more precisely describing heat transport in living bodies.

In the present model, a sphere of radius *b* = 0.01 m is filled with a homogeneous medium (*e.g.*, a biological simulant or tissue phantom) populated with magnetic NPs evenly distributed in space, which act as a space- and time-dependent heat source (note that the heating power *P*_in_ is explicitly dependent on the local, instantaneous temperature, so that it turns out to be a function not only of time but also of the distance from the sphere's centre, even if the NP distribution is uniform in space).

Here, the only important mechanism of heat generation by magnetic nanoparticles is assumed to be Néel's relaxation,^72^ Brown's relaxation being negligible at the frequency of operation (*f* = 1 × 10^5^ Hz).^72,73^ Moreover, in real living tissues or phantoms other types of energy dissipation (such as the ones derived from NP translational motion) are almost completely hindered.^72,74,75^

**Fig. 4:**
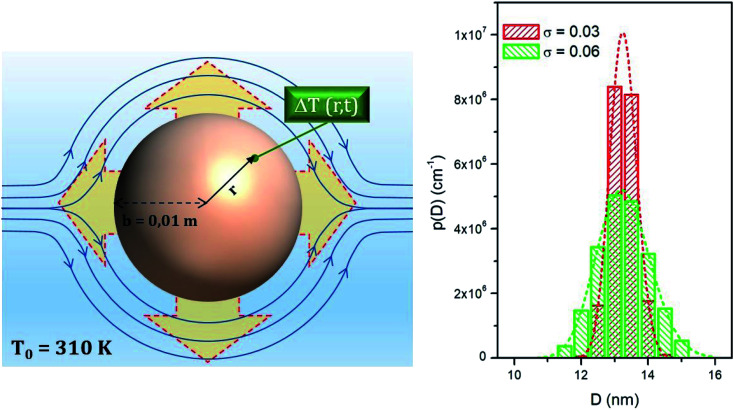
Left panel: sketch of the heating model with convective boundary conditions. The sphere is made of a phantom containing a uniform dispersion of magnetite nanoparticles. Right panel: lognormal probability density functions used in this study, reported both as histograms at intervals of half a nanometer and as continuous curves (dashed lines).

The thermal model analyzed here is shown in the left panel of Fig. 4.

The heated medium is immersed in a continuously flowing fluid representing blood (initially at the temperature *T*_0_), and the temperature is found by numerically solving an equation appropriate to a medium with uniform thermal conductivity and thermal diffusivity:4
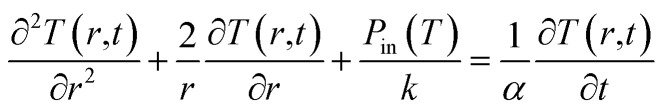
where *T*(*r*,*t*) is the local, instantaneous temperature inside the sphere, *P*_in_(*T*) is the heating power of the homogeneous NP distribution, and *k* and *α* are the phantom's thermal conductivity and thermal diffusivity. Their values, *k* = 0.5 W m K^−1^ (applicable to real phantoms^76,77^) and *α* = 1.4 × 10^−7^ m^2^ s^−1^, are considered to be constant owing to the limited temperature increment above *T*_0_ considered in this paper.

The loss of heat in the medium is taken into account by introducing an appropriate boundary condition, *i.e.*, by assuming that the heat exchange at the sphere/fluid boundary occurs by convection at local blood's temperature and is determined by the process of tissue-blood perfusion, an effect which greatly varies from tissue to tissue.^35,78^ The boundary condition is:5
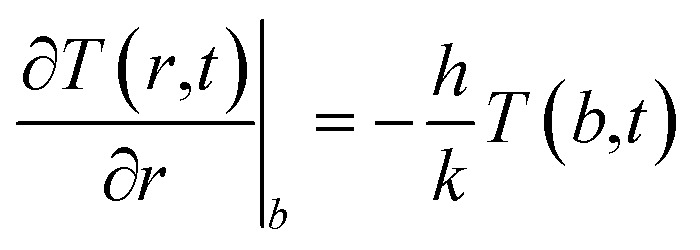
where *h* is the convective heat transfer coefficient. The value used here is *h* = 133 W m^−2^ K^−1^, corresponding to a blood perfusion coefficient as high as 4 × 10^4^ W m^−3^ K^−1^, typical of tumor tissues.^78^

The approach to the steady-state temperature is determined by the thermal parameters *k* and *α* of the phantom; however, the magnitude of the input power *P*_in_, which is remarkably influenced by *y*, has an effect not only on the steady state temperature itself, as predictable, but also on the initial slope of the Δ*T*(*b*/2,*t*) curve: the larger *y* is, the higher is the initial slope.

### 2.6 Effect of nanoparticle size distribution

Nanoparticle systems used in typical applications are never ideally monodisperse. The effect of particle size distribution is however easily accounted for when particles are non-interacting. In this paper, the size distribution is modelled using a lognormal probability density function, whose mode has been fixed at *D* = 13.25 nm whilst the variance *σ* takes the values *σ* = 0.03 or *σ* = 0.06. Histograms representing the two distribution functions at intervals of half a nanometer are shown in the right panel of Fig. 4, along with the corresponding continuous functions.

The distributions obtained using either value of *σ* describe in a realistic manner actual systems of magnetite nanoparticles for magnetic hyperthermia. All the size-averaged quantities are obtained as discrete weighted sums of the results for monodisperse systems using diameter intervals of half a nanometer.

**Fig. 5:**
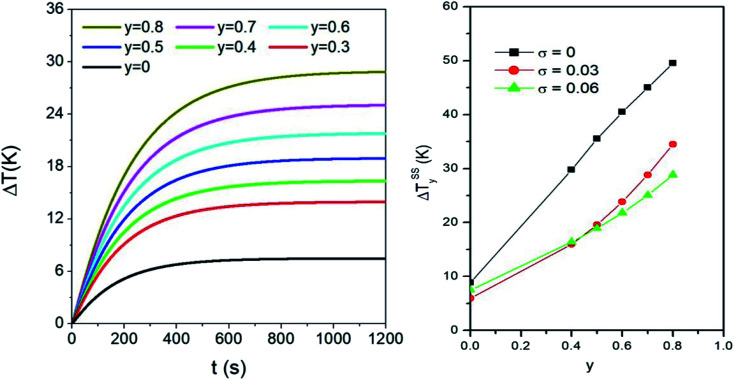
Left panel: effect of the taper parameter *y* on the time evolution of temperature in *r* = *b*/2, for a sample containing a volume fraction *f*_V_ = 0.5% of polydisperse magnetite nanoparticles (*σ* = 0.06). Right panel: effect of the taper parameter on the steady state temperature Δ*T*^SS^_*y*_ achieved in a monodisperse system with *D* = 13 nm and in two polydisperse systems with the same *σ* values as in the right panel of Fig. 4.

An example of application of eqn (4) and (5) in a sample containing polydisperse particles is given in the left panel of Fig. 5. There, the time evolution of the temperature increment Δ*T* = *T* − *T*_0_ halfway between the sphere centre and the boundary (*r* = *b*/2) is shown for different values of the taper parameter *y*. The magnetic NPs are distributed in size according to the lognormal with *σ* = 0.06.

When nanoparticles are distributed in size, the variance *σ* and the taper parameter *y* have combined effects on the steady-state temperature, as shown in the right panel of Fig. 5. Generally speaking, the final temperature increases with increasing *y*. However, the values of Δ*T*^SS^_*y*_ are larger in the monodisperse system than in the two polydisperse systems: this can be explained considering that the heating power of nanoparticles is the largest for the value of *D* considered here (13 nm). In polydisperse systems, adding the weighted contributions from particles of different sizes necessarily lowers the final temperature reached when only particles with *D* = 13 nm are present. On the other hand, it is observed that doubling the value of *σ* has minor consequences on the Δ*T*^SS^_*y*_ values. The differences between the two curves reflect the complex interplay between the heating power of nanoparticles (and its temperature dependence) and the relative weight of each diameter *D* in the *p*(*D*) distribution.

## 3 Optimization of magnetic hyperthermia treatments

Trapezoidal driving-field waveforms can be applied to optimize hyperthermia treatments based on the use of magnetic nanoparticles. Three possible goals of high relevance in the therapeutic practice will be discussed here, along with the corresponding experimental procedures.

(a) An important instance in hyperthermia treatments is to be able to finely tune the thermal efficiency of nanoparticles and to quickly modify the steady-state temperature of a treated region, when it turns out to be slightly incorrect with respect to the initial aim, and to do so without the need of interrupting the healing treatment.

(b) Subjecting a tumor tissue to a short high-temperature treatment followed by a more prolonged heating at a lower temperature could be of interest in view of the possible advantages derived from the combination of ablation and hyperthermia processes within the same curing treatment, *i.e.*, a greater therapeutic efficacy of the apoptosis process together with a reduction of tissue inflammation typically caused by the necrosis process.^79^

(c) Another important issue is the reduction of the overall time taken for a typical hyperthermia treatment. Thermal inertia of the region subjected to heating often results in rather long initial transients, therapeutically mostly useless, which amount to non-negligible fractions of the total treatment time. A handy method to drastically reduce the heating transient would be beneficial in the therapeutic practice.

When a treatment of magnetic hyperthermia is implemented using a sinusoidal driving field, there is in practice no way to easily achieve the outlined objectives. In contrast, trapezoidal driving-field waveforms are sufficiently versatile to allow an user to reach all the aforementioned goals, as described in the following paragraphs.

**Fig. 6:**
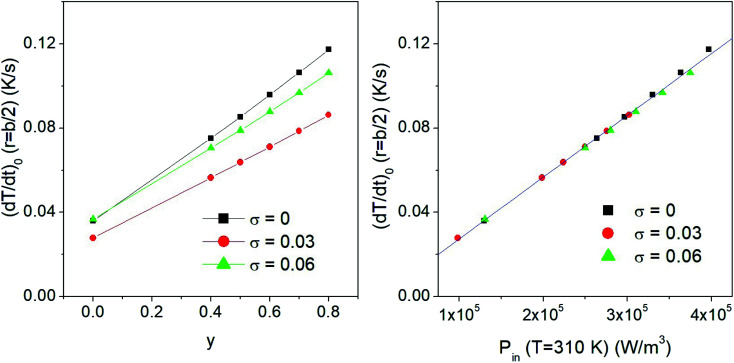
Left panel: effect of taper parameter *y* on the initial value of the time derivative of temperature in *r* = *b*/2, in samples containing either monodisperse (*D* = 13 nm) or polydisperse nanoparticles. Right panel: correlation between the initial time derivatives for different *y* values (as reported in the left panel) and the corresponding heating power *P*_in_ generated by the nanoparticles at the starting temperature, *T* = *T*_0_.

It has to be stressed that in all the examined cases a key role is played by the taper parameter. In fact, the initial slope of the heating curve *T*(*r*,*t*) turns out to linearly depend on *y*, making the heating curves steeper and faster obtained using a higher *y*. This is shown in the left panel of Fig. 6, where typical results for a monodisperse system with *D* = 13 nm and two polydisperse systems with *σ* = 0.03 and *σ* = 0.06 are reported. The volume fraction of the nanoparticles is *f*_V_ = 0.5%. The initial derivative of the heating curve, 
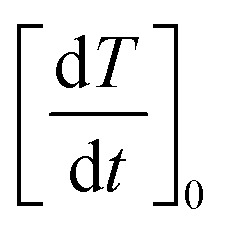
, has been evaluated in *r* = *b*/2 between *t* = 0 and *t* = 10 s, where the temperature still varies linearly. In all the cases a direct proportionality of the initial slope with *y* is observed.

These results also show that the width of the size distribution function plays a remarkable role; in particular, a non-trivial behaviour of 
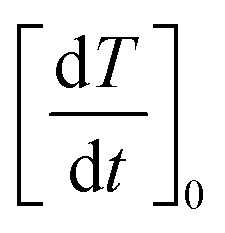
 with *σ* is observed. This result can be explained considering that the key parameter determining the initial slope of the heating curve is the value of the input power *P*_in_ at *T* = 310 K, which depends on both *y* and *σ*. When the values of the time derivative at different *y* are plotted as functions of the corresponding values of *P*_in_(*T* = 310 K), a single master curve is obtained, as shown in the right panel of the same figure.

All the results reported in the following examples refer to the volume fraction *f*_V_ of the nanoparticles corresponding to 0.5% of the total volume of the spherical sample. The magnetic nanoparticles are distributed in size according to a lognormal law with *σ* = 0.06 and are subjected to a driving-field of frequency *f* = 1 × 10^5^ Hz, with a vertex field of 100 Oe (≈8 × 10^3^ A m^−1^).

### 3.1 Fine tuning of the steady-state temperature

**Fig. 7:**
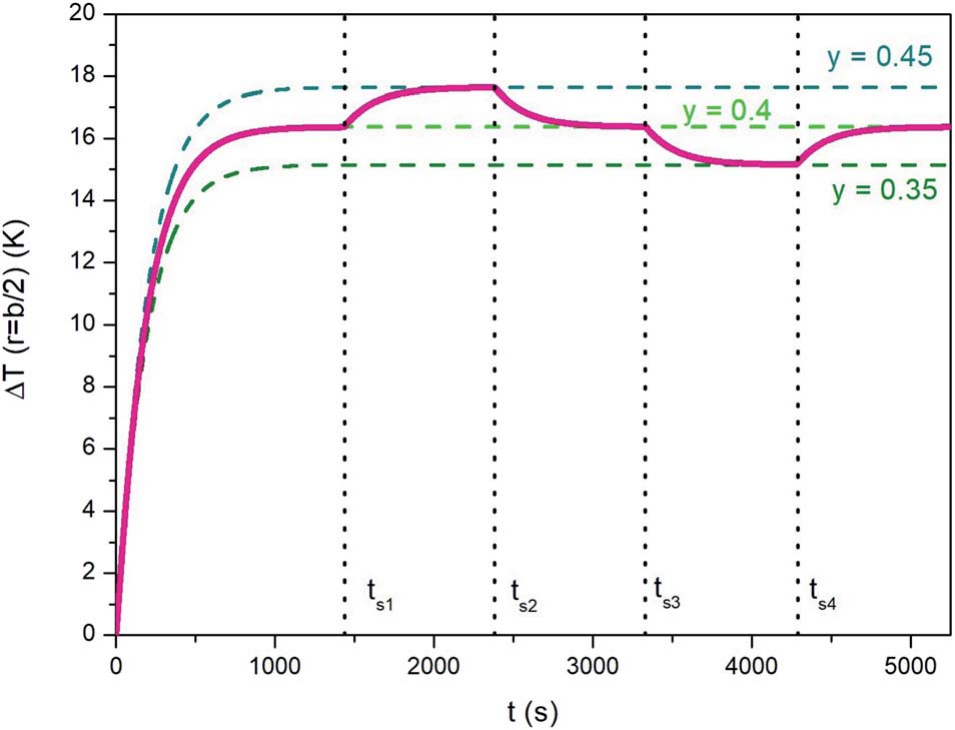
Full line in magenta: temperature–time curve showing how the steady state temperature of a heated medium can be adjusted by acting on the taper parameter of the trapezoidal waveform. See text for details.

Using a trapezoidal wave of intermediate taper parameter *y* is a good starting point if one looks for the ability to vary the steady-state temperature of the heated medium. The value *y* = 0.4 has been used to generate the heating curve shown in Fig. 7 (the full line shows the temperature increment Δ*T* above *T*_0_ for *r* = *b*/2). Once the steady-state temperature corresponding to *y* = 0.4 has been reached (dotted light green line in Fig. 7), it can be easily modified (in both directions) by simply changing the parameter *y*; this can be done by suitably changing the ac voltage applied to the inductive circuit (see Section 2.3). The effect is illustrated in Fig. 7; the taper parameter is suddenly increased or decreased at the switching times *t*_si_ reported in the figure, leading the system's temperature to change by about one kelvin in absolute value. The steady-state temperatures corresponding to *y* = 0.45 and to *y* = 0.35 (dotted dark green lines in Fig. 7) are reached after a transient which is basically determined by the intrinsic parameters *k* and *α* of the phantom. We refer to this thermal behaviour as the “natural” response of the medium, in contrast with the forced response described below, point c).

Therefore, small changes of the taper parameter lead to small increments or decrements of the steady-state temperature, precisely as required if its value turns out to be not optimal, *i.e.*, different from the target temperature.

Of course, tuning the steady state temperature could be possible – at least in principle – using a standard sinusoidal waveform. For instance, the final temperature can be modified either by changing the driving frequency or by changing the vertex field. Both solutions are however unpractical and not easily controllable (a change of either parameter has non-linear effects on the steady-state temperature of the sample). In contrast, changing the taper parameter *y* is a much easier task and does not require changing the driving-field frequency or amplitude. Only the amplitude and width of the pulsed driving voltage are to be adjusted in a controlled manner (see Section 2.3).

### 3.2 Initial overheating of a treated region

**Fig. 8:**
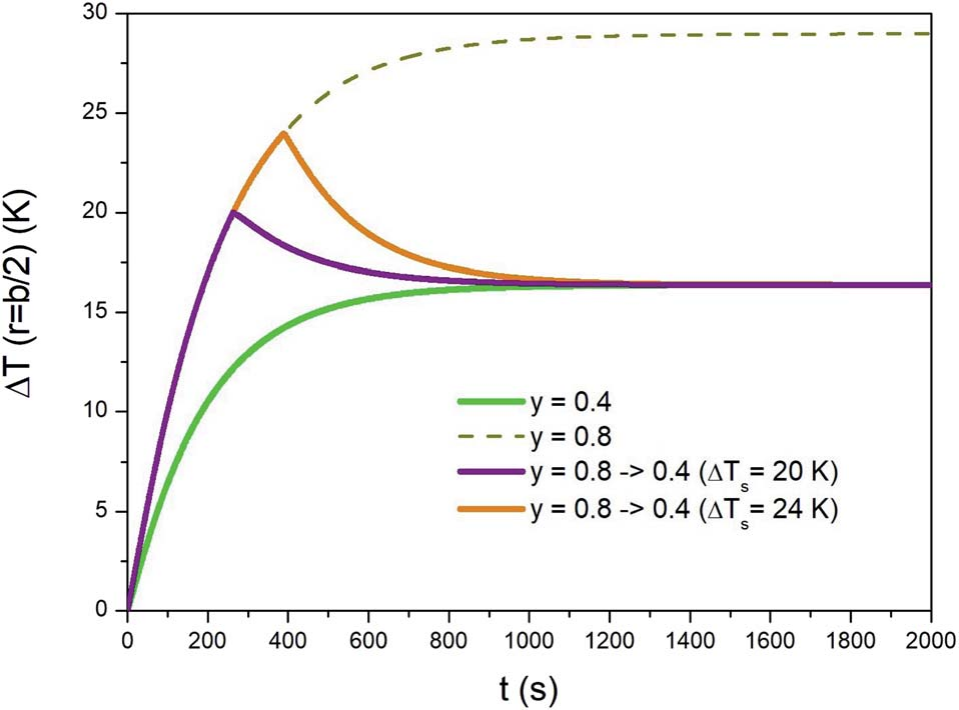
Temperature–time curves showing how a sample can be overheated for a short time at the beginning of a prolonged isothermal treatment. See text for details.

An initial overheating of the treated region above the standard temperature of treatment can be achieved by switching the taper parameter from a high value to a lower one during the initial transient. A typical example is shown in Fig. 8, where the evolution of temperature Δ*T* is again studied in *r* = *b*/2. It is supposed that the steady-state temperature is the one obtained by applying a trapezoidal waveform with taper parameter *y* = 0.4 (green line). The overheating can be achieved by applying a waveform of a considerably higher taper parameter (*e.g.*, *y* = 0.8; dotted line in Fig. 8) and by switching the waveform down to *y* = 0.4 once a previously appointed temperature has been reached. Two examples corresponding to different peak temperatures are shown in the same figure. It can be noted that when *y* is suddenly decreased from 0.8 to 0.4 the temperature quickly departs from the Δ*T*(*b*/2,*t*) curve (dotted line). The time of cooling is determined by the thermal parameters of the phantom. The time taken by the medium to reach the steady state is basically the same with and without the initial overheating.

### 3.3 Active reduction of thermal transients

**Fig. 9:**
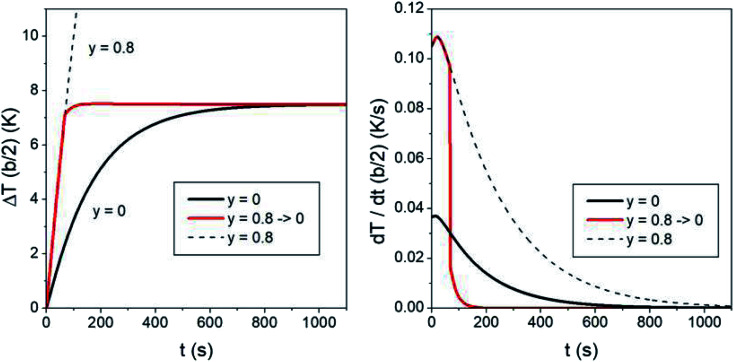
Left panel: temperature–time curves showing how the initial thermal transient can be reduced with respect to the standard case of a harmonic/triangular waveform. Right panel: time derivatives of the curves reported in the left panel. See text for details.

The characteristic time needed to reach the steady-state temperature is basically determined by the thermal parameters *k* and *α* in the heat equation and by the size of the heated region. Changing the fraction of magnetic nanoparticles only has minor effects on the thermal transients (for a constant heating power *P*_in_ the temperature transient is completely independent of the nanoparticle fraction;^80^ however, in the present case *P*_in_ depends on temperature, so that the duration of the transient is weakly modified by the number of nanoparticles per unit volume also). Actually, the duration of the temperature transient is an aspect of primary importance in *in vivo* application of hyperthermia, because during a part of this time the temperature of the treated region is too low to produce therapeutic effects. The question arises if it is possible to substantially reduce the duration of the initial transient. Again, trapezoidal waveforms display their versatility. In fact, acting on the taper parameter of a trapezoidal waveform provides a natural approach to speed up the hyperthermia treatment by shortening the initial transient time. The effect is shown in Fig. 9 (the time evolution of the temperature increment Δ*T* is always evaluated in *r* = *b*/2). The black line in the left panel shows the Δ*T*(*b*/2,*t*) curve using a symmetric triangular waveform (*y* = 0). In this case the initial transient's duration is of about 700 s (≈12 minutes) before the steady-state temperature Δ*T*^SS^_*y*=0_ is reached.

Now, if the heating procedure is initiated with a trapezoidal waveform of a high taper parameter (*y* = 0.8 has been used here), the temperature of the medium rises at a much faster rate, as previously discussed. If a sudden switch of the taper parameter down to *y* = 0 is operated once a prefixed temperature has been reached in the sample (in the present case, 95% of Δ*T*^SS^_*y*=0_), the red line shown in Fig. 9 is obtained (the dotted line shows the curve which would be obtained without operating the switch). Now, the temperature very quickly reaches Δ*T*^SS^_*y*=0_, as further indicated in the right panel of the figure, where the time derivatives of the two curves are reported. It can be checked that in this way the duration of the heating transient is reduced by about 80% with respect to that of the standard case, leading to a substantial improvement of the healing efficacy using the same treatment time.

**Fig. 10:**
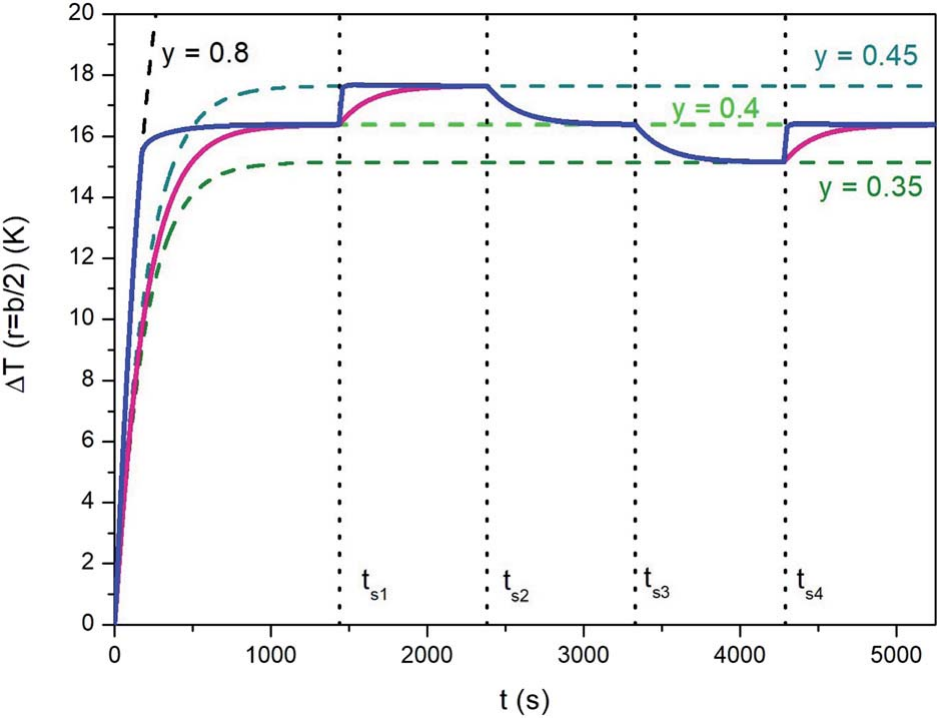
Blue full line: temperature–time curve showing how the time needed by temperature adjustments can be reduced for the same study case analyzed in Fig. 7. The line in magenta corresponds to the “natural” response of the sample. See text for details.

A similar technique can be exploited to speed up the temperature adjustments discussed in Section 3.1 and illustrated in Fig. 7. The effect of repeatedly changing *y* is shown in Fig. 10, where the blue line has been obtained by modifying the taper parameter of the applied waveform (the line in magenta referring to the “natural”, *i.e.*, not forced case studied in Section 3.1 is reported for comparison). The proposed procedure involves the following steps:

- Initial temperature rises with *y* = 0.8;

- switch from *y* = 0.8 to *y* = 0.4 when Δ*T* has reached 95% of the steady-state temperature for *y* = 0.4 (Δ*T*^SS^_*y*=0.4_); the new value of *y* is maintained until *t* = *t*_s1_;

- when *t* = *t*_s1_, *y* is switched to 0.8 and the temperature starts increasing at a very high rate, so that the corresponding steady-state temperature is approached very rapidly (in fact, the blue line becomes a nearly vertical segment);

- switch from *y* = 0.8 to *y* = 0.45 when Δ*T* has reached 95% of the difference between Δ*T*^SS^_*y*=0.45_ and Δ*T*^SS^_*y*=0.4_; the new value of *y* is maintained until *t* = *t*_s2_;

- when *t* = *t*_s2_, *y* is switched down to 0 (triangular waveform);

- sudden switch from *y* = 0 to *y* = 0.4 when the decreasing temperature Δ*T* reaches 5% of the difference between Δ*T*^SS^_*y*=0.45_ and Δ*T*^SS^_*y*=0.4_; the new value of *y* is maintained until *t* = *t*_s3_.

A similar procedure (with obvious adaptations) is followed in the subsequent steps.

Therefore, fine tuning of the steady-state temperature can be remarkably sped up, at least if Δ*T*^SS^ needs to be increased. Downward temperature adjustments are not sped up by the present technique with respect to the “natural” case shown in Fig. 7: changing *y* has virtually no effect on the time constants of cooling.

As a final remark, the steady-state temperature increments reported for the three examples we have just discussed are typically higher than the ones used in the therapeutic practice of magnetic hyperthermia aimed at malignant cell apoptosis. Actually, the aim of the paper is to outline the features of the proposed technique rather than to provide a recipe to reach a given temperature. All the reported thermal effects can be obtained starting from (or ending with) any steady-state temperature; every result is easily rescaled by simply changing a single experimental parameter, such as, the volume fraction of magnetic nanoparticles dispersed in the medium.

## 4 Conclusions

Using RF magnetic fields of a trapezoidal rather than sinusoidal waveform allows substantial improvements to be gained in therapeutic applications involving hyperthermia from magnetically activated nanoparticles. A trapezoidal waveform can be easily generated and tuned and is versatile enough to control the target temperature by acting on a single parameter, *i.e.*, the taper parameter *y* of the trapezoidal wave.

Changes of the taper parameter have been shown to produce changes in the hysteresis loops of a system of non-interacting magnetite nanoparticles, and therefore in their SLP. These effects have been tested using a simple heating model that simulates a region of living tissue exchanging heat with the surroundings through forced convection carried out by tissue-blood perfusion.

It has been shown that fine tuning of the working temperature of a magnetically heated region can be achieved by suitably acting on *y* without implementing any other changes. In this way, the working temperature can be suitably adjusted in real time. The relationship existing between the taper parameter and steady-state temperature can be exploited to obtain controlled overheating of the treated region, which can allow combined ablation-hyperthermia treatments to be performed. Moreover, the direct proportionality between the taper parameter and initial slope of the temperature–time curve can be exploited to considerably reduce the initial temperature transients.

Using a trapezoidal RF magnetic field poses no particular harm to the safety of healthy tissues crossed by the magnetic flux lines. Of course, trapezoidal waveforms are characterized by a higher rate of change of the RF driving field than a harmonic waveform of the same amplitude and frequency. Such a rate is inversely proportional to (1 − *y*); however, the values of taper parameter *y*, driving-field amplitude *H*_V_ and magnetizing frequency *f* considered in this paper are such that detrimental effects on healthy tissues can be safely excluded.

In conclusion, a substantial optimization of the heating performance of magnetic nanoparticles where Néel's relaxation plays a dominant role can be achieved not only by developing better nanomaterials, but also by increasing one's ability to efficiently extract the power released by a nanoparticle system.

The effects described in this paper provide a glimpse of the benefits of using specifically tailored driving-field waveforms to activate the magnetic nanoparticles. A further step towards application will be the development of an experimental and metrological framework devised to validate the magnetic and thermal model and requiring:

- The design of a resonant circuit equipped with a solenoid coil able to generate a homogeneous radio-frequency magnetic field of a sufficient amplitude, trapezoidal waveform and tunable taper parameter;

- the design of an experimental setup to perform accurate measurement of space- and time-resolved temperature (using, *e.g.*, a set of fiber-optical thermometers), duly taking into account the non-adiabatic conditions of the heated system and the various mechanisms of heat exchange with the surrounding environment;

- use of biological simulants and phantoms, characterized by dielectric and thermal properties close to those of living tissues, in order to obtain reliable estimates of the relevant heat transport mechanisms.

These steps towards validation of the proposed method will have to be complemented with additional know-how to secure a smooth transfer to *in vitro* and finally *in vivo* experiments. In particular, the knowledge of the physical properties is to be integrated with biological/biomedical skills and competences: biocompatibility tests, evaluation of magnetic nanoparticle biodistribution in living tissues, and measurements of the thermal and conductive properties of bloodstream have to be carefully performed.

However, we point out that directly acting on the efficiency of heat generation from magnetic nanoparticles brings about significant advantages expected to remain basically unchanged even in complex experimental arrangements or in applications involving living bodies.

## Conflicts of interest

There are no conflicts to declare.

## Supplementary Material
